# The potential of genomic epidemiology: capitalizing on its practical use for impact in the healthcare setting

**DOI:** 10.3389/fpubh.2025.1504796

**Published:** 2025-01-31

**Authors:** Nicole Pacchiarini, Caoimhe McKerr, Mari Morgan, Thomas R. Connor, Christopher Williams

**Affiliations:** ^1^Communicable Disease Surveillance Centre (CDSC), Public Health Wales, Cardiff, Wales, United Kingdom; ^2^Public Health Genomics Programme, Public Health Wales, Cardiff, Wales, United Kingdom; ^3^School of Biosciences, Cardiff University, Cardiff, Wales, United Kingdom

**Keywords:** SARS-CoV-2, COVID-19, whole genome sequencing (WGS), healthcare acquired infections, *Clostridioides difficile*, nosocomial infection, sequencing

## Abstract

The rapid detection and containment of healthcare-associated infections (HCAIs) is critical in preventing and controlling infectious disease outbreaks within healthcare settings. Whole genome sequencing (WGS) has emerged as a powerful tool for tracking the transmission dynamics of pathogens and when used alongside traditional epidemiological methods it can better inform our understanding of the pathogen origin, pathway and extent of transmission. Additionally, WGS can aid in identifying previously unrecognized reservoirs of infection, allowing for more effective control strategies and targeted interventions. This article describes the incorporation of WGS into infectious disease management in Wales and explores it in the context of COVID-19 and *Clostridioides difficile*. We also describe the developments made to the workforce in Wales to enable the expansion of WGS and reflect on the resources, infrastructure and training frameworks still required.

## 1 Introduction

Whole genome sequencing (WGS) of pathogens allows tracking of mutations across populations, enabling mapping of isolate relatedness and supporting detailed understanding of disease spread. Identifying differences in genomics sequence or variant trends over time also allows better understanding of pathogen transmission dynamics across populations. With contextual data, we can then consider strain and sequence diversity in an environment to better understand pathogen origin, incidence, and risk factors, potentially improving outbreak control (1). We describe and discuss the incorporation of WGS into infectious disease management in healthcare settings in Wales, and adaptations to the workforce that have allowed this. Wales is a country within the United Kingdom with ~3.2 million inhabitants and an area of 21,218 square kilometers (2). Management and strategic development of health and social care services sits with the Welsh parliament (Senedd) and Welsh Government. The centralized public health agency [Public Health Wales (PHW)], created by Welsh Government, operates most of Wales' microbiological diagnostic laboratories. Epidemiology and laboratories are brought together in a single organization, allowing a joined-up genomics strategy which ensures equitable coverage and service delivery across the country.

## 2 COVID-19

In the two first years of the SARS-CoV-2 pandemic (Apr 20–Apr 22), over 800,000 COVID-19 patients were admitted to UK hospitals (3). The COVID-19 pandemic exacerbated specific healthcare-associated infections (HCAIs) (4–6) challenges, presenting unique logistic issues due to its wide clinical disease spectrum and inconsistent control measures, most without a current evidence base (7). HCAIs continue to cause considerable burdens of illness and mortality in vulnerable populations, even when community infection prevalence is lower (8, 9). Swift HCAI detection and management are crucial to prevent onward transmission to susceptible patients and staff.

Genomic analysis is rapidly becoming crucial to revealing insights into hospital transmission pathways: hospitalized COVID-19 cases are both community and healthcare acquired, and genomics can help guide different approaches to control (10). Over the SARS-CoV-2 pandemic, the value of pathogen genomics was demonstrated globally. Wales played a leading role in early work utilizing WGS: PHW sequenced their first case on 06/03/2020; by October 2020, >7,500 samples had been sequenced and utilized in real-time to support the response (11). This information was used at every level—from patient management in immunocompromised individuals to national-level analysis to detect population trends alerting to new variant emergence (12–14). More widely, studies throughout the pandemic evidenced that inclusion of genomic analyses revealed transmission links, guide interventions, such as staff screening, and supported tailored patient pathways (7, 15–18).

During this time, a Wales exercise based on meta-analyses of studies investigating SARS-CoV-2 ancestral strain infections was undertaken to establish variations in incubation period. In line with the literature, new Delta variants demonstrated shorter incubation periods than ancestral strains (15, 19–21). This information allowed re-evaluation of the contemporaneously accepted nosocomial acquisition case definitions, supporting flexibility of approaches during times of rapid pace and evolving understanding. The centralized microbiological and epidemiological service delivery within PHW ensured data could be rapidly utilized —in service and for research—in response to need.

Moreover, routine genomic surveillance can assist in detection of asymptomatic cases in outbreaks. This provides a better idea of outbreak size and scale and prompts changes to testing schedules and infection prevention and control (IPC) practices (22). In one prospective study, weekly genomic sample reporting identified transmission events associated with “green” areas, where no symptomatic cases had been identified (8). The resulting information was used to directly inform operational decisions, such as patient placement and staff movement. These examples highlight the role genomic data can lend to operational decisions.

## 3 *Clostridioides difficile*

The advent of next generation sequencing within PHW under the Anaerobe Reference Unit (ARU) and the Pathogen Genomics Unit (PenGU) has transformed understanding of in-hospital *Clostridioides difficile* (23). The *C. difficile* Genomics Sequencing and Typing (DIGEST) service has been active since 2018, receiving ISO 15189 accreditation in 2020—one of the first services of its type globally to achieve this milestone (24). This collective has strengthened understanding of the course and characteristics of *C. difficile* incidence and enabled exploration genomic data's utility within clinical scenarios. Genomic data has already supported examinations of evolutionary rate (23) and reservoir diversity (25), and we are linking genomic and epidemiological data to understand transmission in healthcare settings. The work includes toxin negative cases, allowing inclusion of a broader pool of infections to better understand transmission.

In October 2022, a period of increased incidence was observed in inpatients of a North Wales hospital (26). Surveillance data, combined with sequencing results, revealed that 22 of 40 cases were genomically closely related and likely a discrete outbreak, and 18 cases had unique sequences. Univariable analysis suggested that outbreak cases were approximately six times more likely to be healthcare rather than community acquired (OR 5.8; *p* = 0.02). No other variable was statistically significant, but outbreak cases were more likely to die, have had recent healthcare interventions, and less likely to have had toxin positive first tests (27). In this outbreak, genomic data distinguished within hospital outbreak cases from patients with community acquired sporadic strains, allowing the response to focus on specific high-risk areas, which positively impacted IPC efforts and case finding. High numbers of toxin negative results demonstrated the usefulness of typing all isolates regardless of toxin status and the importance of IPC interventions for toxin negative patients, which is reinforced in some of the literature (28–30).

## 4 Workforce implications

Alongside laboratory and bioinformatics services, two recently established PHW teams have been essential for facilitating and maximizing the practical use of genomics data. The Genomic Epidemiology Unit was established early 2020, sitting within the Wales Communicable Disease Surveillance Center (CDSC). The team consists of experienced epidemiologists who utilize sequencing technologies in diagnostics, surveillance and outbreak investigations. The Healthcare Epidemiology Network was established in 2019, with national surveillance center epidemiologists embedded within local health board IPC and microbiology teams. Prior to creation of these teams, results of routine sequencing were communicated directly to clinicans and/or microbiology teams, which limited their use for broader surveillance and epidemiological purposes. These two successful models have enabled more refined surveillance loops and improved synthesis of epidemiological and genomic data to inform prevention and control activities (31).

In 2020, the ARU began sending details of genomically indistinguishable samples within health boards to local healthcare epidemiologists and microbiologists. In March 2022, the PHW Genomic Epidemiology Unit extended this work through development of processes to detect and flag cross-health board clusters. This alerting process to the Healthcare Epidemiology Network highlights strains/sequences within health boards, while also flagging where cases may have originated from or spread to an area outside the health board. Outputs include epidemiological summaries and projects to standardize definitions of signals and triggers for action. This directly supports more nuanced undertstanding of infections and transmission pathways, evidencing the mixture of community and healthcare associated cases in hospital settings. Ongoing collaborations between IPC teams and genomic epidemiologists have been strengthened since 2020, contextualizing hospital outbreaks and providing insights into how community prevalence impacts hospital environments (27). The model has now evolved to encompass the Genomic Epidemiology and Healthcare Epidemiology teams as core parts of incident investigations as standard, allowing that specific expertise to be included in discussions from the outset.

As HCAI rates increase in Wales (32), alongside concerns regarding antimicrobial resistance among common microorganisms (33, 34), the role of the modern epidemiologist offers specific training and skill in surveillance and evaluation, research, and outbreak management and preparedness. Suitably trained infectious disease epidemiologists embedded in infection prevention programmes offer a solid cornerstone for utilizing and harnessing the power of genomic data. Having focused professionals with expertise in interpreting and acting upon genomics information closely aligned with local areas is a strength of the system; this enables the uses of genomic data to be tailored to local need (35). A central team providing a similar service would not have the relationships and local knowledge required to fully understand signals in the data, and to ensure rapid responses. The key limitation is the limited resilience of working through a small network, with a single healthcare epidemiologist for a health board. Further, because detailed understanding of genomics is not part of routine epidemiologist training, developing these skills in healthcare epidemiologists requires careful planning (31). The challenge of mainstreaming genomics into routine practice has been recognized across healthcare systems and is a major barrier to development of teams such as the Healthcare Epidemiology Network. Although not a standard or established role in health organizations in the UK, the Healthcare Epidemiology Network and dedicated Genomic Epidemiology team have demonstrated huge value in Wales, particularly during the COVID-19 pandemic.

## 5 The future of genomic epidemiology in healthcare

In Wales, ongoing collaborations between IPC teams and genomic epidemiologists have strengthened the public health response by providing a means to critique and synthesize research and practice and distill information to audiences unfamiliar with epidemiological and molecular principles. During the COVID-19 pandemic, the addition of genomics data to rule out transmission events was critical to develop ward specific control measures, based on learnings from settings where outbreaks had or had not been contained (36). Interventions included introducing regular testing of asymptomatic individuals, operational discussions around exclusion/restriction of staff movement, provision of additional personal protective equipment, and later, further vaccination efforts. Multi-disciplinary teams that integrated laboratory and testing expertise alongside IPC teams and genomic epidemiologists, enabled design and delivery of end-to-end services scoped to fit the needs of public health professionals on the acute end of responding to incidents.

Investment in rapid sequencing technologies and streamlined data integration is crucial for maximizing utility of genomic epidemiology. However, there is still some way to go before genomic epidemiology can reach its full potential in impacting real-time operational decisions. It is crucial that it is prioritized and given resource, with continued close collaboration between guidance bodies and frontline infection preventionists (7). Automation of data processes using electronic patient records, can enhance timeliness and accuracy of genomic surveillance (21). Logistical frameworks need to be well considered, including resource in laboratories, bioinformatics and accurate methods of feeding into surveillance systems. Development of a trained workforce with epidemiolgical skill in the application of pathogen genomics, is a core component of the Genomics Delivery Plan for Wales. These coherent and collegiate models support sustainability of innovation in genomic surveillance activities, and investment in the future workforce (31).

Disease surveillance combined with sequencing data requires considerable resource and infrastructure investment for routine use within IPC practice. While phylogenetic models can evaluate sequence data and phylogenetic history, challenges remain in incorporating timely patient metadata. Wales benefits from linked systems (e.g., a national pathology information management system), however, challenges exist in joining systems and managing/cleaning data to enable use in larger-scale analyses. Incomplete information and mistakes (e.g., transcription errors) create significant barriers to utilization of data available through PHW established platforms. Linking these data in real-time can help IPC teams focus intervention to have most impact. Such measures are likely to improve patient health and prove cost saving in the future (37).

## 6 Discussion

As we re-examine pandemic preparedness post-COVID-19, integration of genomics with traditional epidemiological methods offers a path forward. We have shown that addition of genomic epidemiology into IPC practices represents an important step toward more effective and informed infectious disease management. Genomic data should play an increasingly important role in shaping future outbreak responses, ensuring that healthcare settings are better equipped to prevent and control infections. Collaboration between guidance bodies and frontline IPC teams is essential for developing evidence-based strategies that balance infection control with operational functionality.

Finally, for continued advancement of these activities, it is vital to ensure that our workforce is appropriate to meet future needs and expansions of the genomics service. The needs of pathogen genomics need to be integrated into workforce planning (35). As genomics becomes a more significant tool in infectious disease, health protection and microbiology as areas of clinical practice, ensuring staff are equipped with training to fully utilize genomic data is essential. Throughout the pandemic, the genomics team worked closely with IPC and health protection colleagues to support interpretation of genomic data. Weekly advisory sessions and support for development and interpretation of reports ensured data was used and understood. Over time, as their skills and understanding improved, staff became more confident, resulting in more autonomous operation. Mainstreaming genomics across the healthcare system is key and is the focus of activity at an operational level within PHW, as well as part of larger strategic work including the Welsh Genomics Workforce Strategic Plan (November 2024). Development of genomics capabilities requires provision of support for service establishment and specific consideration regarding training for clinical staff and epidemiologists, to produce the next generation of genomics-aware scientists.
